# The Weierstrassian movement patterns of snails

**DOI:** 10.1098/rsos.160941

**Published:** 2017-06-07

**Authors:** Andy Reynolds, Giacomo Santini, Guido Chelazzi, Stefano Focardi

**Affiliations:** 1 Rothamsted Research, Harpenden AL5 2JQ, UK; 2 Dipartimento di Biologia, Università di Firenze, Via Madonna del Piano, 6, 50019 Sesto Fiorentino, Italy; 3 ISC-CNR, 50019 Sesto Fiorentino, Italy

**Keywords:** Lévy flight foraging hypothesis, intertidal molluscs, limpets, chaos, behavioural plasticity, *Patella*

## Abstract

Weierstrassian Lévy walks are the archetypical form of random walk that do not satisfy the central limit theorem and are instead characterized by scale invariance. They were originally regarded as a mathematical abstraction but subsequent theoretical studies showed that they can, in principle, at least, be generated by chaos. Recently, Weierstrassian Lévy walks have been found to provide accurate representations of the movement patterns of mussels (*Mytilus edulis*) and mud snails (*Hydrobia ulvae*) recorded in the laboratory under controlled conditions. Here, we tested whether Weierstrassian Lévy walks and chaos are present under natural conditions in intertidal limpets *Patella vulgata* and *P. rustica,* and found that both characteristics are pervasive. We thereby show that Weierstrassian Lévy walks may be fundamental to how molluscs experience and interact with the world across a wide range of ecological contexts. We also show in an easily accessible way how chaos can produce a wide variety of Weierstrassian Lévy walk movement patterns. Our findings support the Lévy flight foraging hypothesis that posits that because Lévy walks can optimize search efficiencies, natural selection should have led to adaptations for Lévy walks.

## 1. Introduction

Lévy walks are a popular but controversial model of forager movement patterns [1–4]. They comprise clusters of many short steps with longer steps between them. This pattern is repeated across all scales with the resultant clusters creating fractal patterns that have no characteristic scale. The hallmark of a Lévy walk is a distribution of step lengths with a heavy power-law tail; *p*(*l*) ∼ *l*^−*µ*^ with 1 < *µ* ≤ 3, where *l* is the step length and *µ* is the power-law (Lévy) exponent (‘∼’ means distributed as). The hallmarks of Lévy walks have, to some extent, been observed in the molecular machinery operating within cells [5], bacteria [6,7], T cells [8], a diverse range of marine predators[9–11], mussels [12,13], mud snails [14,15], honeybees and bumblebees [16,17], midge swarms [18], the wandering albatross and shearwaters [19–21], human hunter–gatherers [22], and have even been observed in trace fossils—the oldest records of animal movement patterns [23]. Their occurrence is often attributed to the execution of an advantageous searching strategy in accordance with the Lévy flight foraging hypothesis (LFFH) that posits that because Lévy walk can optimize search efficiencies, natural selection should have led to adaptions for Lévy walks [1,2]. The key to understanding does, however, lie with the elucidation of the underlying generative mechanisms. Many simple putative generative mechanisms have been identified [3]. But these findings tend to challenge the LFFH, rather than support it, because they suggest that Lévy walks arise freely from seemingly benign or innocuous behaviours. This in turn suggests that their optimality, if it occurs, is fortuitous rather than the result of natural selection.

Mud snails (*Hydrobia ulvae*) and mussels (*Mytilus edulis*) appear to be exceptions [12–15], since the explanation of their movement patterns can be connected to the LFFH. These species appear to approximate optimized Lévy walk searching patterns as tri-modal walks, i.e. by continuously switching between three different modes of walking, each with its own distinctive average step length [13–15]. The close resemblance with an optimized Lévy walk searching strategy is indicative of selection because it seems to require that the switching rates and average step lengths be finely tuned. Tri-modal walks with arbitrarily chosen parameters will typically not resemble any kind of Lévy walk.

The mud snails' tri-modal walk is, in fact, a truncated form of Weierstrassian Lévy walk [13]. Weierstrassian Lévy walks can be characterized by a hyper-exponential step-length distribution

1.1 $$p(l)=\frac{q-1}{q}\sum_{j=0}^{\infty }q^{-j}b^{-j}L^{-1}\exp\left(\frac{-l}{b^{j}L}\right).$$

Note that a step drawn from an exponential distribution with mean length *Lb^*j*^* is *q* times more likely than is a step drawn from an exponential with the next longest mean. As a consequence, a walker will typically make a cluster of *q* steps with mean length *L* before making a step of mean length *bL*, and so initiating a new cluster. About *q* such clusters separated by a distance of about *bL* are formed before a step of mean length *b*^2^*L* is made and so on. Eventually, a hierarchy of clusters within clusters is formed. This scale-free pattern is the hallmark of a Lévy walk. The construction given in equation (1.1) therefore provides a recipe for approximating Lévy walks as multi-phasic walks, an approximation that becomes ever more precise as the number of modes (number of terms included in the summation) increases.

Mud snails and mussels have movement patterns consistent with the first three hierarchical levels in Weierstrassian Lévy walks [13,15]. Truncation inevitably introduces characteristic scales that make movement patterns scale finite. But unlike other finite-scale movement patterns variability around the characteristic scales is huge and self-similar, and so movement patterns can retain the hallmarks of Lévy walks over a broad range of scales [13].

The movement patterns of mussels [12] and mud snails [14] were recorded in the laboratory under controlled conditions. A key open question is whether Weierstrassian Lévy walks and chaos are present under natural conditions and so fundamental to how foragers interact and experience the world. Here, we addressed this question by testing for the prevalence of Weierstrassian Lévy walks and chaos under natural conditions, in the presence of environmental stimuli, in intertidal limpets *Patella vulgata* and *P. rustica*. We then show that the programming for these Lévy walk movement patterns does not need to be very sophisticated or clever on the organism's part, as these movement patterns can be a by-product of chaos. Many other putative biologically plausible mechanisms have been identified for the generation of Lévy walk movement patterns [3], but in contrast with the chaotic pathway these tend to produce just one kind of Lévy walk rather than a variety of Lévy walks. This is a crucial distinction because plasticity is a prerequisite for the LFFH.

## 2. Foraging behaviours of intertidal snails

Movement patterns of intertidal snails are highly specific adaptations to the alternation of favourable and unfavourable nictemeral and tidal phases. Intertidal snails are compelled to rest in refuges (often home scars) during unfavourable phases to avoid predation and/or physical stress (e.g. dehydration) and to move to graze on the algal film only when external conditions are favourable [24,25]. For example, the limpet *P. vulgata*, an important algal grazer of northeast Atlantic rocky shores, is often active during nocturnal low tides and after each foraging excursion it returns to its home scar, although considerable variability has been observed [26]. The limpets dwelling in the highest shore fringe perform shorter excursions than those living in the lower fringe, and size-related variability was also observed [27,28]. *Patella rustica*, on the other hand, live in the upper shore fringe of the cliffs of the weakly tidal Mediterranean Sea and are only active when the upper shore is well splashed by waves during storms [29].

## 3. Material and methods

### 3.1. Study areas and data collection

*Patella rustica* were monitored at Cala Galera (Italy) in autumn (8–11 November) 1988 and summer (8–9 July) 1989 during stormy weather. *Patella vulgata* were studied at Menai Bridge, Wales, UK in April and November 1992. Following Della Santina *et al*. [27], we distinguish between ‘high shore’ (HS) limpets, zoned between 4.65 and 5.0 m above the level of the lowest low tide and ‘low shore’ (LS) limpets, zoned between 3.9 and 4.4 m above the level of the lowest low tide.

**Figure 1.**
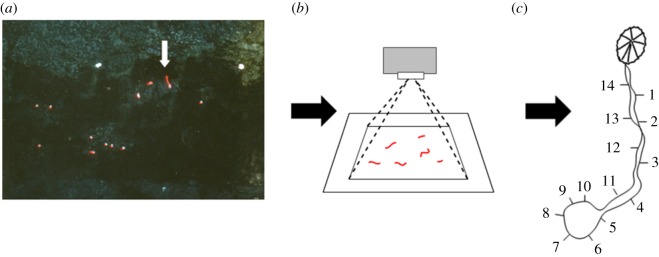
A schematic of data acquisition. (*a*) Example of a 10 min exposure to record *P. vulgata* movements. The movements of limpets appear as continuous red lines (white arrow). (*b*) All the pictures, shot at regular time intervals, are then projected in sequence and (*c*) the tracks of all limpets are reconstructed and digitized. The numbers represent the ending points of all the movement segments observed in (*a*). Knowing the time of start and ending of each exposure, it is possible to attribute a time to each point along the track.

Limpets from both populations were monitored using a motographic method described in full detail by Chelazzi *et al*. [30]. A light emitting diode and a battery were glued to the shell and the position of the animal was recorded by an automatic camera (Hasselblad 500 EL) placed on a column facing the limpets resting positions. The camera was remotely controlled to shoot pictures at regular time intervals. Each exposure lasted 5 or 10 min (5 *P. vulgata* November; 10 *P. rustica* and *P. vulgata* April, see below) and thus recorded the entire path covered by a limpet during that time. Individual paths were graphically reconstructed from pictures and stored into a computer as sequential files of coordinates with reference to an arbitrary origin, using a digitizer (figure 1).

Recordings of *P. vulgata* behaviour were carried out during nocturnal low tides, as previous investigations have clearly demonstrated that at this location limpets are only active during low tide [27]. Limpets were monitored during four and five nocturnal low tides in April and November, respectively. The activity of *P. rustica* were continuously monitored for several days but the periods a limpet spent inactive (i.e. not moving) on its home scar were not considered in the analysis. These excursions usually lasted 4–6 h. More details on data acquisition and preparation for *P. vulgata* and *P. rustica* can be found in Della Santina *et al*. [27] and Chelazzi *et al*. [31]. We recorded the movement of 15 and 14 *P. rustica* in November and July, respectively, while for *P. vulgata* 17 and 18 animals were monitored in November and April.

### 3.2. Analysis of movements

Each foraging excursion is represented by a set of locations, or fixes, with respect to an arbitrary origin on the shore and known temporal coordinates. Owing to the rigid homing behaviour of the two *Patella* species examined in this study, an excursion is defined as a movement trajectory starting and ending on the same resting site (the home scar). Movement trajectories were digitized without any *a priori* fixed step length, to represent as accurately as possible limpets movements ([30] for details). For each individual, we then aggregated regularly sampled snail tracks into sequences of ‘steps’. This was done using the approach of Humphries *et al*. [19] in which the movement patterns are first projected onto the *x*- and *y*-axes to create two one-dimensional movement patterns for each individual. Humphries *et al*. [19] showed that the projection of a Lévy walk is itself a Lévy walk and that projection does not result in non-Lévy walks being misidentified as Lévy walks and vice versa. Turns in these projections can then be identified in an unambiguous way as occurring where the direction of travel changes. Without projection, turns can only be identified by making reference to arbitrarily defined critical-turning angles.

The minimum step length was taken to be 1 cm unless stated otherwise. We find that our results to do not change significantly when this minimum length ranges between 0.5 and 5 cm (electronic supplementary material, §S1). The step-length distributions were fitted to tri- or four- modal exponentials corresponding to truncated Weierstrassian Lévy walks and to competing models (exponential, bi-exponential and power-law distributions). Power laws are indicative of true Lévy walks, exponentials are null models of the movement patterns and bi-exponentials are indicative of bi-modal searching. Fittings were performed by maximum-likelihood methods [32] and the best model distribution was identified using the Akaike information criterion [33].

Here, following Clauset *et al*. [32], the absolute goodness-of-fits (GOF-test) of the model distributions were quantified by *p*-values. If the *p*-value is large, then the difference between the empirical data and the model distribution can be attributed to statistical fluctuations alone; if it is small, then the model distribution is not a plausible fit to the data. Following Clauset *et al*. [32], we make the relatively conservative choice and reject the model distribution of interest if *p* ≤ 0.1, otherwise it is accepted as being plausible.

Two different analyses were performed. Population level analysis refers to different sets defined by the variable ‘species’ (*P. rustica* and *P. vulgata*), season and zonation (HS and LS, for *P. vulgata* only). We pooled all steps of the individuals in each population set. Second, we investigated the movement patterns of individual limpets. This was done for *P. vulgata*, by pooling all excursions (usually 4–5) made by each individual. We also tested for the presence of chaos in the time series. Since this kind of analysis is data intensive, it was only done for the population sets. It is generally accepted that a unique intrinsic and observable signature of systems exhibiting deterministic chaos is a fluctuation power spectrum with an exponential frequency dependency [34–36]. White noise processes (i.e. Poisson processes and all other processes which have no temporally correlated behaviour) and multi-phasic walks (electronic supplementary material, §S2), on the other hand, have flat spectra while ‘*1*/*f*’ noise (found in scale-invariant systems with long-range correlations) have spectra with power-law frequency dependency [37].

The fluctuation power spectrum is calculated from the extracted one-dimensional turns which define a time-series *u*(*t*). If, for example, turns occurred at times *t* = 3Δ*t*, 5Δ*t*, 6Δ*t*, … ,(*N *− 2)Δ*t*, *N*Δ*t*, where Δ*t* is the time interval between consecutive positional fixes defining the steps and *N* is the total number of these positional fixes then the entries in the time-series *u*(*t*) would be 0, 0, 1, 0, 1, 1, … ,1, 0, 1. The power spectrum of *u*(*t*), *S*( *f*), is the square of the magnitude of the Fourier transform of *u*(*t*) and is given by

3.1 $$S(\;f)={\left|\frac{1}{\sqrt{2\pi }}\sum_{k=0}^{N}u(t){\text{e}}^{-i2\pi ft}\right|}^{2}\equiv \frac{1}{2\pi }F(\;f)F^{*}(\;f),$$

where *t *= *k*Δ*t* is the time at which the *k*th positional fix was made; *f* is frequency, *F*( *f*) is the discrete Fourier transform of *u*(*t*) and F*( *f*) is its complex conjugate. The data were neither smoothed nor tapered prior to transforming. Spectra were fitted to stretched exponentials, $S(\;f)\propto {\text{e}}^{-{\left(\;f\text{/}f_{0}\right)}^{\nu }}$ and to power laws 1/*f^ µ^* using maximum-likelihood methods. Good fits to stretched exponentials would be consistent with the presence of chaos, poor fits would be indicative of the absence of chaos. We performed an independent test for chaos by calculating the largest Lyapunov exponents (electronic supplementary material, §S3).

The detection of chaos in the time-series data allows us to distinguish between ‘Lévy-like statistics’ in the movement pattern data, and ‘Lévy walk behaviour’ *per se*; that is, to distinguish between step-length distributions displaying power-law scaling over a range of scales (which does not account for autocorrelation) and independent and identically distributed step lengths following distributions displaying power-law scaling. A multi-phasic walk would be a Weierstrassian Lévy walk if and only if the step-length distribution were a finite mixture model, i.e. if at each step the snails ‘chose’ independently from the different components of the power-law mixture distribution. Alternatively, if the behaviour is better described by a hidden Markov model, then the snail would move consistently with a characteristic step length for number of steps.

## 4. Results

**Figure 2.**
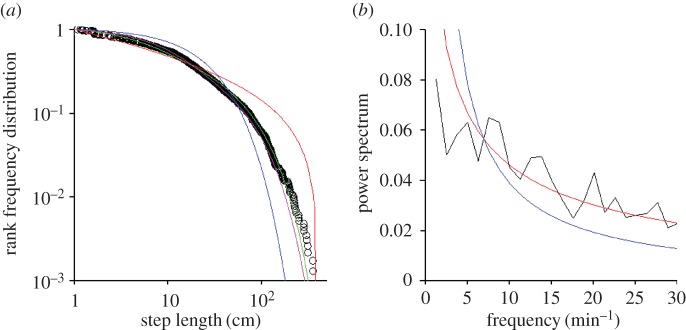
(*a*) Movement patterns of *P. vulgata* at Menai Bridge, UK. Rank frequency plot of the tested model step-length distributions (exponential, blue; bi-exponential, pink; four-mode Weierstrassian Lévy walk, green; and power law, red) and empirical data (open circles). (*b*) Power spectrum (black line) together with the best fit stretched exponential (red line) and the best fit power law (blue).

At the global level, our movement pattern data for *P. vulgata* closely resemble a four-mode Weierstrassian (GOF-test, *p* = 0.135) (figure 2*a*). We find no support for our data being either exponential, bi-exponential or power-law distributed (table 1). The four-mode Weierstrassian fit to our data is, however, only slightly better than the three-mode Weierstrassian fit. Note also that our step lengths are practically independent (electronic supplementary material, §S4).

**Table 1.** Model selection for pooled *P. vulgata* data.

model	AIC	ΔAIC	Akaike weight
four-mode Weierstrassian	18888.0	0.0	0.97
three-mode Weierstrassian	18895.0	7.0	0.03
bi-exponential	18953.8	65.8	0.00
Power law	19275.5	387.5	0.00
exponential	19611.1	723.1	0.00

Our data are also indicative of the presence of chaos (figure 2*b*), as the power spectrum is well represented by a stretched exponential and less well represented by a power law which overestimates the low-frequency content of the power spectrum and underestimates the high-frequency content. Positivity of the largest ‘Lyapunov exponent’ is also indicative of the presence of chaos (electronic supplementary material, §S3).

**Figure 3.**
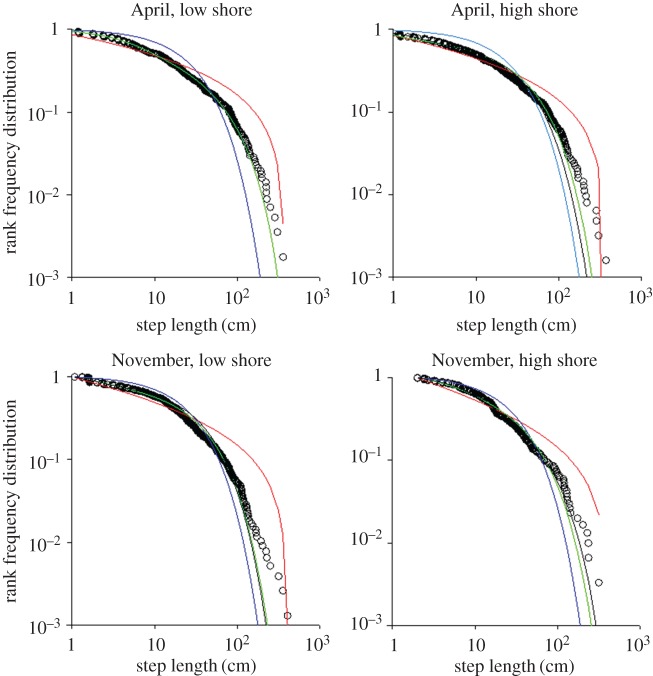
Movement patterns of *P. vulgata* at Menai Bridge, UK. Rank frequency plot of the tested model step-length distributions (exponential, blue; bi-exponential, black; three-mode Weierstrassian Lévy walk, green; and power law, red) and empirical data recorded (open circles) in April and November and different zones (high shore and low shore) of the cliff where the home scar was located. For the November, high shore case the minimum step length was 2 cm rather than 1 cm because there were no steps shorter than 1.5 cm.

The movement patterns of most of the studied individuals are well fitted by Weierstrassian Lévy walks in all but one of the ecological conditions studied (table 2). The rank-frequency plots of the different models are reported in figure 3 and clearly show a very good fit of the three-mode Weierstrassian Lévy walk in the four ecological situations. The presence of a rarely occurring fourth mode which was evident in the globally pooled data is not evident when the data is subsampled. There were *fewer* long steps observed than predicted by the fitted distribution. Indeed, comparing figures 3 and 5 one may note that the excess of short steps is much more common for *P. vulgata* than *P. rustica*. Our interpretation is that the former species is severely constrained by the tidal phase and is compelled to return to the scar, and so the number of longer movements is reduced. The latter is instead free in its movement during a storm which can last several days. Nonetheless, the GOF-test is always highly non-significant (April: LS, *p* = 0.567; HS, *p* = 0.165, November: LS, *p* = 0.50; HS, *p* = 0.380).

**Table 2.** Model comparisons for individual movement patterns of *P. vulgata* recorded in April and November at Menai Bridge, UK, and different zones (high shore and low shore) of the cliff where the home scar was located. The best model is highlighted in italics.

		AIC weight					
individual	steps	power law	exponential	bi-exponential	three-tier WLW	*μ* (from power-law fit)	*μ* (from WLW fit)
*November: low shore*							
08	145	0.00	0.00	0.16	*0.84*	1.09	1.07
09	172	0.00	0.00	0.01	*0.99*	1.13	1.33
53	119	0.00	0.00	0.06	*0.94*	1.21	1.21
55	66	0.00	0.00	*0.91*	0.09	1.21	1.14
59	70	0.00	0.00	0.21	*0.79*	1.33	1.24
60	63	0.00	0.00	0.14	*0.86*	1.07	1.21
62	85	0.00	0.00	0.32	*0.68*	1.20	1.17
63	105	0.03	0.00	0.04	*0.97*	1.22	1.19
64	99	0.00	0.00	0.12	*0.88*	1.16	1.22
*November: high shore*							
01	76	0.00	0.00	0.30	*0.70*	1.35	1.25
05	164	0.01	0.00	0.01	*0.98*	1.23	1.17
11	34	0.00	0.00	0.24	*0.76*	1.62	1.62
14	115	0.00	0.00	*1.00*	0.00	1.54	1.33
52	108	*0.53*	0.00	0.00	0.47	1.34	1.32
56	34	0.01	0.00	*0.50*	0.49	1.78	1.43
65	100	0.00	0.00	0.27	*0.72*	1.86	1.56
67	48	0.00	0.00	*1.00*	0.00	1.67	1.38
*April: low shore*							
02	108	0.00	0.00	0.08	*0.92*	1.10	1.31
03	85	0.00	0.00	0.11	*0.89*	1.02	1.71
04	25	0.00	0.34	0.18	*0.48*	1.02	1.28
08	88	0.00	0.00	0.14	*0.86*	1.09	1.50
09	123	0.00	0.00	0.32	*0.68*	1.05	1.38
15	32	0.02	0.00	0.09	*0.89*	1.18	1.07
16	103	0.00	0.00	0.06	*0.94*	1.09	1.46
*April: high shore*							
01	77	0.36	0.00	0.00	*0.64*	1.20	1.11
05	93	0.00	0.00	0.04	*0.96*	1.08	1.16
06	86	0.01	0.00	0.00	*0.99*	1.23	1.11
10	61	0.00	0.00	0.18	*0.81*	1.07	1.76
11	38	0.00	0.00	0.36	*0.64*	1.02	1.37
12	56	0.01	0.00	0.30	*0.69*	1.04	1.40
13	103	0.00	0.00	0.01	*0.99*	1.13	1.06
14	56	0.00	0.00	0.08	*0.92*	1.13	1.06
17	29	*0.38*	0.00	0.30	0.32	1.20	1.30
18	7	0.06	*0.47*	0.15	0.31	1.12	1.07
19	18	0.15	0.01	0.34	*0.49*	1.00	1.09

There is variability in the average *μ* values observed in the four environmental conditions, illustrated by a pooled analysis and confirmed by the analysis of single limpets (table 3). In April, limpets perform more meandering foraging excursions in the LS than in the HS (Student's *t*-test with Satterthwaite correction d.f. = 183.5, *t *= −8.04, *p* < 0.0001) while the reverse holds in November (Student's *t*-test with Satterthwaite correction d.f. = 150.58, *t* = 6.37, *p* < 0.0001).

**Table 3.** *Patella vulgata* at Menai Bridge, UK. For different periods and zones we report the results of the analysis where all steps were pooled (pooled analysis) and where we investigate individual movement (individual analysis). Pooled analysis: median value of the Lévy exponent, *μ*, for the best fit Weierstrassian Lévy walks together with 95% confidence limits derived by a bootstrap with 100 replications. Individual analysis: number of studied individuals and the Lévy exponent, *μ*, for the best fit Weierstrassian Lévy walk (from table 2).

sampled areas		pooled analysis			individual analysis	
period	height	*μ* median value	95% CI		*N*	*μ* ± s.e.
April	high	1.17	1.09	1.28	11	1.23 ± 0.07
	low	1.43	1.24	1.50	7	1.38 ± 0.06
November	high	1.29	1.22	1.41	7	1.38 ± 0.05
	low	1.15	1.10	1.20	9	1.20 ± 0.02

**Figure 4.**
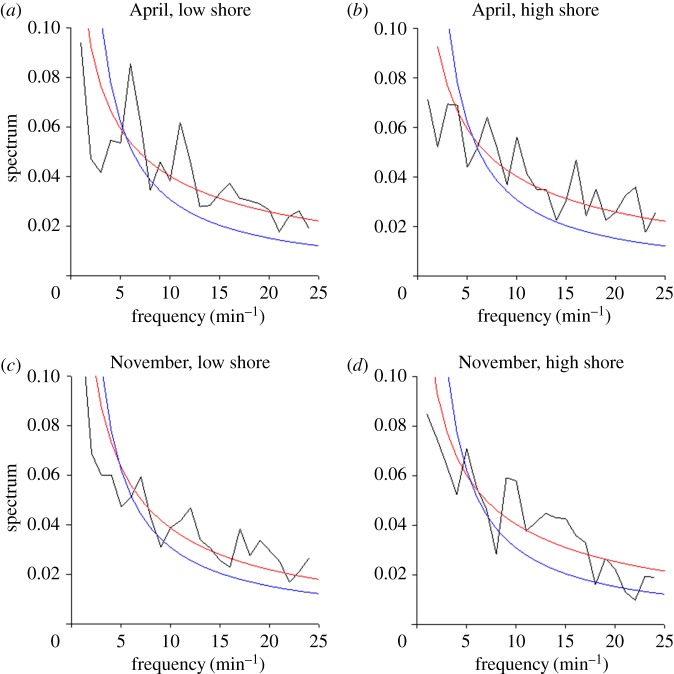
Movement patterns of *P. vulgata* at Menai Bridge, UK recorded in April and November and different zones (high shore and low shore) of the cliff where the home scar was located. Power spectrum (black lines) are shown together with the best fit stretched exponentials (red lines) and the best fit power laws (blue lines).

The hallmarks of chaos are evident in the time series of movement collected in the four ecological conditions (figure 4). The power spectra are seen to have exponential rather than power-law frequency dependence. This confirms that the signature of chaos is not an artefact due to the pooling of heterogeneous samples but a systematic feature of these movements.

**Figure 5.**
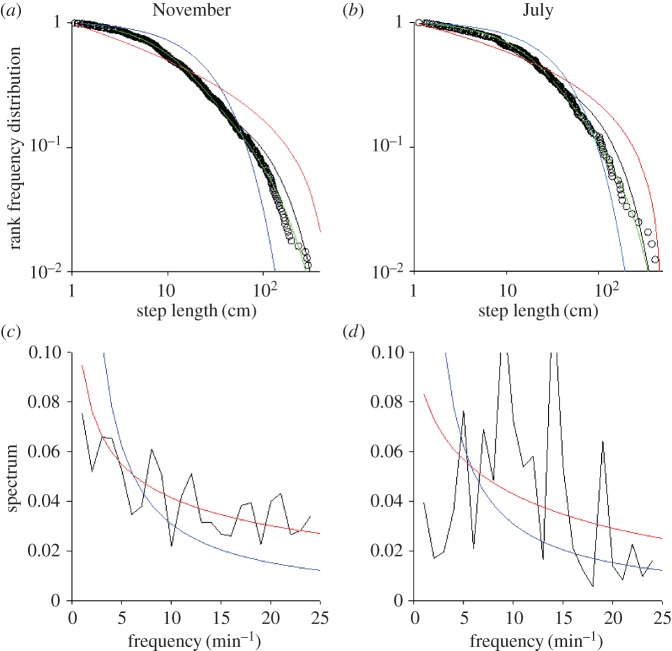
Movement patterns of *P. rustica* at Cala Galera, Italy. Rank frequency plot of the tested models (exponential, blue; bi-exponential, black; three-mode Weierstrassian Lévy walk, green; and power law, red) and empirical data recorded (open circles) in summer and autumn. Power spectrum (black line) together with the best fit stretched exponentials (red line) and the best fit power laws (blue lines).

To test whether or not our results are specific to the population of *P. vulgata* in Menai Bridge we analysed a smaller dataset; a Mediterranean population of the limpet *P. rustica* (figure 5*a*). Even in this species, three-tier Weistrassian Lévy walks fit the experimental data better than do the concurrent model distributions. Indeed, the Akaike weights for Weierstrassian Lévy walks are 1.00 for both seasons and the GOF-test yield *p* = 0.499 and *p* = 0.929 in July and November, respectively. The *µ* values associated with the Weierstrassian Lévy walks are 1.87 and 1.81 in July and November. In both periods, we observed the presence of an exponential spectrum characteristic of a weakly chaotic generator (figure 5*b*). The very noisy aspect of the July plot is probably due to the small number of steps recorded in this period (about 50 per snail, whereas in November we recorded about 200 per snail).

## 5. Chaos can generate Weierstrassian Lévy walks

Lévy walks can be generated in surprising simple, biologically plausible ways and the identification of these processes has gone some way to demystifying the occurrence of Lévy walks in foragers [3]. It is also known that chaos can produce Weierstrassian Lévy walks [38,39]. In the electronic supplementary material, §S5, we show how Weierstrassian Lévy walks arise in a simple chaotic system. The chaotic system examined, a bouncing ball, is chosen because it is easy to comprehend and because the governing equation can be approximated by a prototype model for chaos. Our analysis thereby shows that Weierstrassian Lévy walks can arise for ‘free’ from generic properties of chaos and does not require sophisticated internal *rules* governing the switching between a variety of random walks each with its own characteristic step length.

## 6. Discussion

Our results show that Weierstrassian Lévy walks are common in intertidal snails under different field conditions. This demonstrates that the previously reported occurrence of Weierstrassian Lévy walks in the mud snail *H. ulvae* [14] did not represent a mathematical ‘curiosity’ and may have important impacts on our understanding of the life history of gastropods. It shows that the occurrence of Weierstrassian Lévy walks are not confined to the laboratory as they also arise in natural surroundings in the presence of environmental stimuli. Indeed, we showed such movement patterns occur in two different limpet species, dwelling in quite different intertidal seascapes, in contrasting seasonal periods. We suggested that the occurrence of these Weierstrassian Lévy walks can be attributed to chaos and found evidence for chaos in our movement pattern data. That is, Weierstrassian Lévy walks are just a mathematical consequence of chaos and one which can be realized by the simplest of systems, e.g. a bouncing ball. Nonetheless, this analysis does not locate the source of the chaotic activity, which could arise either intrinsically, in the neural circuits responsible for coordinated movement (central pattern generators) or in sensory systems. Or, the source could be related to the way the limpets respond to subtle environmental cues, or even in the fine-scale structure of the algal biofilms on which the animals graze. The first of these explanations is perhaps more likely because Weierstrassian Lévy walks and chaos have also been observed in molluscs under laboratory controlled conditions [13–15].

We showed (electronic supplementary material, §S5) that the chaotic route to Lévy walking stands apart from the many other potential routes to Lévy walking which have been identified [3] because it produces plastic rather rigid Lévy walks which can be fine-tuned by natural selection in accordance with the LFFH [2]. Nonetheless, it remains to be seen to what extent, if any, the Weierstrassian Lévy walks in snails are adaptive. The values of the characteristic Lévy exponents, *µ*, (tables 1 and 2) are, in fact, significantly different from 2, the value usually associated with optimal searching [2]. Future work could evaluate the impact of Weierstrassian Lévy walks movement patterns on algal gardening and intraspecific competition by limpets [40–42]. It would also be interesting to assess the impact of the persistence of previous trails; trails which could be used by snails to relocate or better to avoid algal patches already exploited in the recent past.

Our results underscore the fact that Lévy walk-like movement patterns can and do arise naturally from the simplest of processes, e.g. chaotic dynamics. They also vividly illustrate that the binary arguments about the relative merits of Lévy walks and multi-phasic walks as models of movement pattern are misconstrued [43], as animals can move in ways that are well approximated by various types of Lévy walks, internally triggered multiphasic walks being but one.

Our analysis has resonance with that of Sims *et al*. [23] who reported that trace fossils from the Eocene demonstrates that ancient movement patterns can be described as hierarchically nested Brownian walk clusters that converge to a truncated Lévy walk. Sims *et al*. [23] remarked that it is striking that the composite Brownian walks were finely tuned to theoretically optimal Lévy walks, suggesting selection pressure for Lévy walk characteristics. Our findings suggest that this congruence is not unexpected given the presence of chaos. Weierstrassian Lévy walks may, therefore, have ancient origins, predating molluscs which are, in fact, one of the most ancient forms of animals that are living amongst us. It would, therefore, be interesting to test explicitly for the presence of Weierstrassian Lévy walks in trace fossils as this was not done by Sims *et al*. [23]. We could then ascertain the origins of Weierstrassian Lévy walks.

## Supplementary Material

Supplementary Material for The Weierstrassian movement patterns of snails by Reynolds et al.

## Data accessibility

Our data and computer codes are deposited with ISC-CNR and can be found at http://wwwold.fi.isc.cnr.it/files/Reynolds_et_al/.
