# Supplementary material for: The Weierstrassian movement patterns of snails
Source: R Soc Open Sci. 2017 Jun 7;4(6):160941. doi: 10.1098/rsos.160941 (PMC5493898; doi:10.1098/rsos.160941)
Supplement: Supplementary Material for The Weierstrassian movement patterns of snails by Reynolds et al. [file rsos160941supp1.docx]

**Supplementary Material for The Weierstrassian movement patterns of snails**

**Andy Reynolds^1*^, Giacomo Santini^2^, Guido Chelazzi^2^ and Stefano Focardi^3^**

^1^Rothamsted Research, Harpenden, AL5 2JQ, United Kingdom. ^2^ Dipartimento di Biologia, Università di Firenze, Via Madonna del Piano, 6, 50019 Sesto Fiorentino, ^3^ISC-CNR, 50019 Sesto Fiorentino, Italy

**1. ROBUSTNESS OF MODEL DISTRIBUTION FITTING**

The relative merits of single exponentials, bi-exponentials, four-mode Weierstrassian LW and power-laws as characterisations of the movement patterns of *P. vulgata* at Menai Bridge do not change when the minimum step-length in our analyses ranges between 0.5 cm and 5 cm (Fig. S1).

**
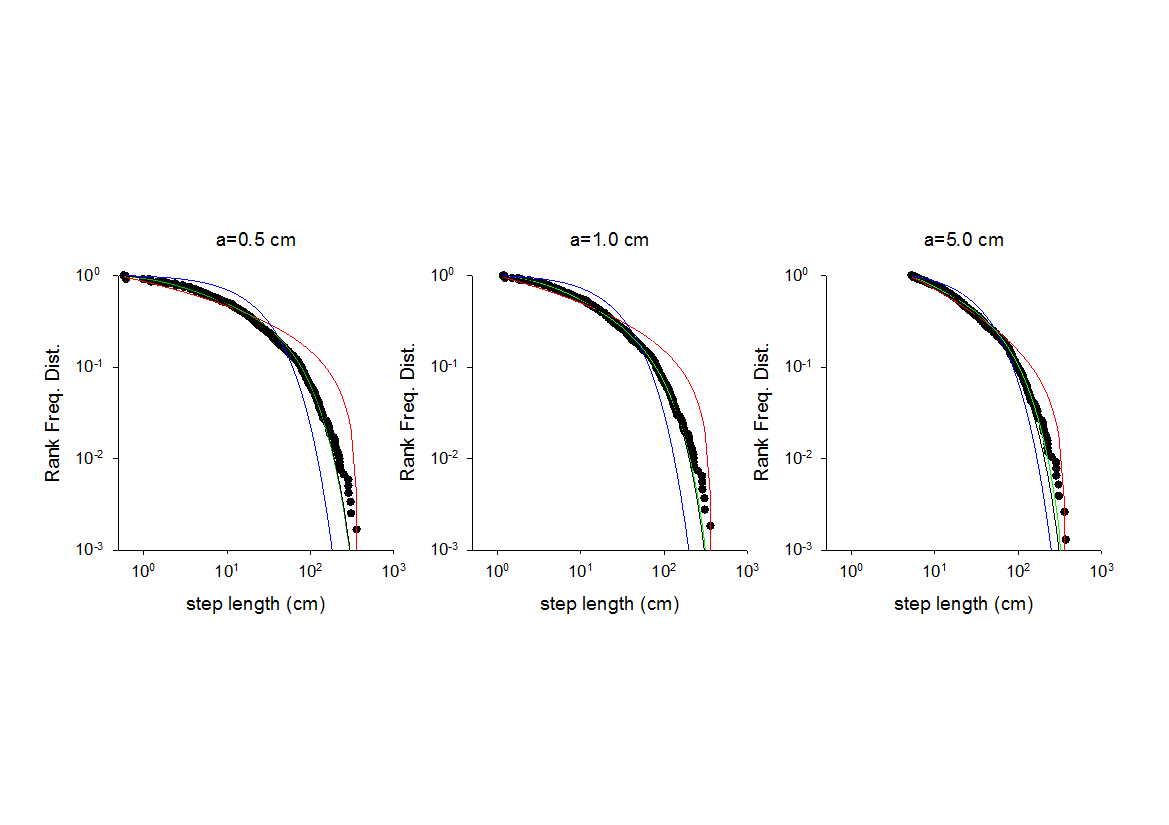
**

**Figure S1.** Movement patterns of *P. vulgata* at Menai Bridge, UK. Rank frequency plot of the tested model step-length distributions (exponential, blue, bi-exponential, black, Weierstrassian LW, green, and power law, red) and empirical data (open circles).

**2. MULTI-PHASIC WALKS HAVE WHITE NOISE SPECTRUM**

We tested whether multi-phasic walks with geometric series in occupancy times and step lengths have spectrum with exponential frequency dependencies, like those characterizing our movement pattern data intertidal limpets. We find that multi-phasic walks have flat (white noise) spectrum (Fig. S2).





**Figure S2.** Ensemble-averaged spectrum for 100 simulated multi-phasic walks each with 100 steps corresponding to 3-tier Weierstrassian Lévy walks with Lévy exponent 2.5, i.e., corresponding to the kind of Weierstrassian Lévy walks found to provide good fits to our movement pattern for intertidal limpets.

**3. FURTHER TEST FOR THE PRESENCE OF CHAOS**

We have further tested for chaos by calculating the largest ‘Lyapunov exponents’. These exponents characterise the separation of initially close *state-space* trajectories. Here they were calculated using the method of Rosenstein et al. [3] which we now outline, as it was fully documented by Rosenstein et al. [3] In this approach the state-space trajectories are expressed as a matrix $U=[U_{1}, U_{2},\ldots,U_{3}]^{T}$where $U_{i}=\left[u_{i},u_{i+1},\ldots,u_{i+(m-1)}\right]$, *i* denotes the time-step and *m=10*, known formally as the ‘embedding dimension’ is effective of the degree of autoregression in the analysis. The algorithm locates the *nearest neighbour* of each point on the trajectory. The nearest neighbour of $U_{j}$ is found by searching for a point,$U_{j}^{/}$, that minimizes the distance to $U_{j}$. This initial distance from $U_{j}$ can be expressed as$d_{j}\left(0\right)=\min_{U_{j}^{/}} \left|U_{j}-U_{j}^{/}\right|$. The largest Lyapunov exponent is then estimated from how the distance,$d_{j}\left(i\right)$, between the pair of nearest neighbour points,$U_{j}$ and $U_{j}^{/}$, grows with time *t=iΔt.* The presence of chaos is indicated by a separation that increases exponentially in time as $d_{j}\left(i\right)\propto e^{\lambda\left(i\Delta t\right)}$ where $\lambda$ is the largest Lyapunov exponent. In practice the presence of chaos is indicated by linear growth of $\left\langle\log d_{j}\left(i\right)\right\rangle$ where denotes the average over all values of *j*. The averaging is crucial when using small, noisy data sets. Note also that the growth of $\left\langle\log d_{j}\left(i\right)\right\rangle$ is expected to saturate at long times since the system is bounded phase space and the average divergence cannot exceed the “length” of the attractor [3].

We conclude positivity of the largest Lyapunov exponents from exponential divergence of initially close state-space trajectories, which provides further support for the presence of deterministic chaos (Fig. S3). Other snails in our sample did not behave in a substantially different way.





**Figure S3** Examples of the average log separation of initially close *state-space* trajectories shows exponential divergence (solid-lines). This divergence indicates that the largest Lyapunov exponent is postive which in turn is indicative of the presence of deterministic chaos (see above). Different colours indicate tracks of four different *P. vulgate* recorded in November. Shown for comparison are two linear fits over times less than 1000 s (dash lines). These fits have R^2^=0.96 and 0.98. The other fits (not shown for clarity) have R^2^=0.92 and 0.97.

**4. AUTOCORRELATION**

In one strict sense Lévy walks are not "uncorrelated", as the autocorrelation function (being the Fourier transform of non-white power-spectrum) is long ranged. From another perspective the autocorrelation function is problematic because step-lengths are non-stationary as the "average" step-length increases over times. This is a manifestation of the scale-free characteristics of Lévy walks [1]. Nonetheless, as illustrated below (Fig. S4) we have attempted to show that the step-lengths in our movement pattern data are not strongly correlated.





**Fig S4**. Here we show the lengths of the n^th^ and n+1^th^ steps. The red line is a linear least squares regression ($l_{n+1}=a+bl_{n}$). The estimate for b=0.065 [95% C.I. 0.030, 0.099] together with r=0.06 indicates step lengths are for practical purposes independent. The F-statistic for the calulated correlation of r=0.06 was F_1,841_=3.56. The probability of getting this F statistic or larger is p=0.06 and so there is no strong evidence in favour of the weak autocorrelation.

The more pertinent question is whether the movement patterns are multiphasic in the sense that long steps are associated with ‘relocation’ phases and short steps are associated with ‘local searching’ phrases triggered by the detection of resources, i.e., whether the movements are extrinsically-cued, or whether they are Lévy walk-like and so intrinsic. This important question was addressed by Kölzsch et al. [2] who showed that the tri-modal movement patterns of mud snails are intrinsically cued.

# 5. WEIERSTRASSIAN LÉVY WALKS IN CHAOTIC SYSTEMS

Here we illustrate that Weierstrassian random walks can arise in a simple chaotic system – a bouncing ball - and show that the key conditions for their occurrence stem from common properties of chaotic systems. The governing equations for our simple chaotic system can, in fact, be approximated by a well-known system of equations, the so-called ‘Standard Map' which is a prototype model for chaos [4]. We thereby identify a potential link between generic properties of chaos and Weierstrassian Lévy movement patterns.

Lévy walks can be generated in surprising simple ways and the identification of these processes has gone some way to demystifying their occurrence in foragers [3]. One of the simplest candidate mechanisms could give rise to Lévy walk movement patterns in ecotonal ecosystems, such as riparian forests where strong environmental gradients force animals to forage within a narrow strip [5]. This restriction would be realised by an animal with straight-line movements, if each time it arrives at an edge of the strip it is ‘deflected’ back at a random angle, $0<\theta<\pi$ drawn from the uniform distribution $p_{\theta}=1/\pi$ (Fig. S5). The horizontal distance travelled along the strip before encountering the opposing edge is $l=L\tan\theta$ where $L$is the width of the strip. The probability density function of these step-lengths is determined by $p_{l}dl=p_{\theta}d\theta$ and so$p_{l}=\frac{1}{\pi}\frac{L}{l^{2}+L^{2}}$. These movement patterns are a Lévy walk with $\mu=2$. This simple analysis makes it clear that Lévy walk movement patterns are not a mathematical abstraction divorced from reality but can instead emerge naturally from seemingly benign, innocuous behaviours and trivial external constraints. It is also illustrative of a general principle, namely that emergent Lévy walk movement patterns tend to be optimized Lévy searches, i.e., be characterized by$\mu=2$. To get a different kind of Lévy walk movement pattern would necessitate inflicting violence on the model. A turning angle distribution $p_{\theta}\propto\tan^{\mu-2}\theta$ would, for example result in Lévy walk with a prescribed Lévy exponent, *μ*, but the modification to the model is too ugly too bear.

Here we show that an analogous situation arises, in part, in deterministic chaos; the key difference being that the resulting Lévy walks are plastic rather than rigid. In this case the forager is deterministic and behaves like a purely elastic billiard ball that travels and bounces without loss of energy, and moves in an ecotone with a rippled edge. One side of the ecotone is flat (at *y*=0) and one side is ribbed (according to *y*=*d+wcos(x)* where *w* is the amplitude of the ripples), as illustrated in Fig. S6A. The scenario is clearly fictitious but is, nonetheless, illustrative of a general principle; namely that chaos can result in plastic Weierstrassian Lévy walks.

The turning angles will depend very sensitively on the incoming angle and on the local curvature of the ripple and has a consequence be widely distributed. This has the potential to lead to Lévy-like movement patterns. Simulated step-lengths (distances travelled between consecutive encounters with y=0) can, in fact, be very well represented by the first three levels of a Weierstrassian Lévy walk (i.e., by the first 3 terms in Eqn. 1), as illustrated in Fig. S6B. This hierarchical structure is readily understood. Most reflection conditions (combinations of incoming velocities and local curvature) lead to short steps, some reflection conditions lead to longer steps, and a few reflection conditions lead to very long steps. This process can be illustrated by Poincaré plots that are records of positions and velocities (Fig. S6C). The tori (the voids embedded in the chaotic sea) in such plots correspond to near repeatable steps and are a defining feature of ‘weak’ chaos [6,7]. The likelihood of making such steps is determined by the size of the tori, and the step-lengths are determined by the average time spent on the tori. Close inspection of the Poincaré sections reveals tori within tori which equates to a hierarchy of typical step-lengths, and so to multi-exponential step-length distributions. If the sizes of the tori and the time-spent on the tori satisfy scaling relations (so that an order of magnitude change in step size is an order of magnitude less likely) then the multi-exponentials (by definition) form a Weierstassian random walk, and so have properties in common with Lévy walks. This frequently happens in ‘weak’ chaos but obviously cannot arise in strong (global) chaos which, by definition, is devoid of tori [6,79].

In weak chaos the structure of the tori and so the resulting Weierstassian Lévy walks are strongly dependent upon system parameters, i.e., the ripple amplitude in the case of billiards, (Fig. S7) and in biological organisms this flexibility equates to plasticity, and so to movement patterns which can be acted upon by selection pressure in accordance with the LFFH.

**

**

**Figure S5** Schematic of the simple mechanism for the generation of optimized Lévy walk searches.

**

Figure S6 A)** Simulation data for the movement pattern of a billiard (red line) within a channel with ripples *y*=1+0.2cos(*x*) (black line). **B)** Cumulative frequency distribution for the step-lengths (o) together with the best fit to a 3-level Weierstrassian Lévy walk, i.e., to the first 3 terms in Eqn. 1. The best fit has *q*=11.3 and *b*=4.25 which corresponds to a Lévy exponent *μ*=2.6. The fitting was obtained using maximum likelihood methods. **C)** A Poincaré section showing successive horizontal positions (modulo π) and horizontal velocities recorded at *y*=0.

**

Figure S7 A)** Simulation data for the movement pattern of a billiard (red line) within channel with ripples y=1+0.11cos(x) (black line). **B)** Cumulative frequency distribution for the step-lengths (●) together with the best fit to a 3-level Weierstrassian Lévy walk i.e., to the first 3 terms in Eqn. 1. The best fit Weierstrassian Lévy walk has q=7.2 and b=2.8 which corresponds to a Lévy exponent μ=2.9. The fitting was obtained using maximum likelihood methods. **C)** A Poincare section showing successive horizontal positions (modulo π) and horizontal velocities recorded at y=0.

**References**

[1] Bouchaud, J-P. &Georges, A. Anomalous diffusion in disordered media: statistical mechanisms, models and physical applications. *Phys. Rep.* **195**, 127-293 (1990).

[2] Kölzsch, A., Alzate, A.., Bartumeus, F., de Jager, M., Weerman, E.J., Hengeveld, G.M., Naguib, M., Nolet, B.A. &. van de Koppel, J. 2015. Experimental evidence for inherent Lévy search behaviour in foraging animals. *Proc. Roy. Soc. B*: **282**, article 20150407 (2015).

[3] Rosenstein, M. T., Collins, J.J. & De Luca, C.J. 1 A practical method for calculating the largest Lyapunov exponents from small data sets. *Physica D* **65** 117-134 (1993).

[4] Méndez-Bermúdez, J.A., de Oliveria, J.A. & Leonel, E.D. A two-dimensional nonlinear map characterized by tunable Lévy flights*.* *Phys. Rev. E* **90**, article:042138 (2014).

[5] Bartumeus, F. et al. Superdiffusion and encounter rates in diluted, low dimensional worlds. *Eur. Phys. J.* **157**,157–166 (2008).

[6] Zaslavsky, G.M. From Hamiltonian chaos to Maxwell's Demon. *Chaos* **5**, 653–661, (1995).

[7] Zaslavsky, G.M., Stevens, D. & Weitzner, H. Self-similar transport in incomplete chaos. *Phys. Rev. E* **48**, 1683–1694 (1993).
